# Application of preoperative computed tomographic lymphography for precise sentinel lymph node biopsy in breast cancer patients

**DOI:** 10.1186/s12893-021-01190-7

**Published:** 2021-04-09

**Authors:** Shishuai Wen, Yiran Liang, Xiaoli Kong, Baofeng Liu, Tingting Ma, Yeqing Zhou, Liyu Jiang, Xiaoyan Li, Qifeng Yang

**Affiliations:** 1 Department of Breast Surgery, General Surgery, Qilu Hospital of Shandong University, Wenhua Xi Road No. 107, Jinan, 250012 Shandong China; 2 Department of Head and Neck Surgery, Fudan University Shanghai Cancer Center, Shanghai, China; 3 Department of Oncology, Shanghai Medical College, Fudan University, Shanghai, China; 4 Department of Radiology, Qilu Hospital of Shandong University, Jinan, China; 5 Pathology Tissue Bank, Qilu Hospital of Shandong University, Jinan, China; 6 Research Institute of Breast Cancer, Shandong University, Jinan, China

**Keywords:** Sentinel lymph node biopsy, Preoperative SLNs and lymphatics localization, Computed tomographic lymphography, CT three-dimensional reconstruction

## Abstract

**Background:**

In light of the extensive application of sentinel lymph node biopsy (SLNB) in clinically node-negative breast cancer patients and the recently investigated failure of SLNB after lumpectomy, it has become important to explore methods for preoperative mapping of sentinel lymph nodes (SLNs) and their lymphatics to direct precise SLNB and improve the identification rate of SLNs.

**Methods:**

Twenty-seven patients with suspected breast cancer based on the results of the clinical examination and imaging were enrolled in the study. Computed tomographic lymphography (CTLG) followed by CT three-dimensional reconstruction was performed to determine the localization of SLNs and lymphatics on the body surface preoperatively. Intraoperatively combined staining with methylene blue and indocyanine green was used to evaluate the accuracy and feasibility of CTLG.

**Results:**

SLNs and lymphatics from the breast were identified using CTLG in all patients, and preoperative SLNs and lymphatics localization on the body surface showed a significant role in the selection of operative incision and injection points. The accuracy rate of SLN and lymphatic detection by CTLG was 92.6% compared with intraoperatively combined staining. Moreover, preoperative CTLG performed well in SLN number detection, and the accuracy rate was 95.2%.

**Conclusion:**

We evaluate the procedure and application of preoperative CTLG in the superficial localization of SLNs and lymphatics, which may lead to a decreased incidence of cutting off the lymphatics of SLNs and consequently more rapid and accurate SLN detection. This method promotes personalized SLN mapping, providing detailed information about the number and anatomical location of SLNs and lymphatics for adequate surgical planning for breast cancer patients.

**Supplementary Information:**

The online version contains supplementary material available at 10.1186/s12893-021-01190-7.

## Background

Breast cancer is the predominant malignancy among women worldwide and accounts for approximately 15% of cancer-related deaths in women [1]. The axillary lymph node (LN) status is essential for the prognosis and treatment of breast cancer patients. Indeed, the 5-year overall survival (OS) rate for breast cancer patients with LN metastasis is reduced by 40% compared to that of patients with negative lymph nodes [2]. Therefore, it is important to determine the lymph node status accurately. Mastectomy and axillary lymph node dissection (ALND) have been widely accepted as the gold standard treatment for patients with breast cancer for decades. Although ALND is a reliable procedure to identify nodal metastasis and maintain locoregional disease control, it leads to notable postoperative complications, including pain, numbness, loss of strength, loss of sensitivity, and edema [3, 4], which significantly affect the quality of life of patients.

Sentinel lymph nodes (SLNs) refer to the initial lymph nodes to receive lymphatic drainage from the primary tumor. SLN is reported to be a predictive factor of metastatic spread to the respective regional nodal basins, and sentinel lymph node biopsy (SLNB) has gradually substituted ALND as the standard surgical procedure for clinically node-negative patients with early-stage breast cancer due to significantly fewer complications and indiscrimination between ALND and SLNB in survival and locoregional disease control [5–11]. With the development of dyes and tracers, various intraoperative detection methods of SLNs have been revealed, including methylene blue dye, indocyanine green fluorescence and radioactive colloid. The combination of radioactive colloid and blue dye, which exhibits a higher SLN detection rate (> 90%) and a lower false negative rate (< 5–10%), is the internationally recommended standard tracer method for SLNB [12]. However, given the risk of exposure to radiation, training of doctors, legislative requirements, the need for professional equipment, and the cost, the application of radioactive colloids in China is limited [13]. On the other hand, the dye method is simple and easy to master after training and is the widely used tracing method for SLNB in China.

For preoperative biopsy, fine needle aspiration cytology (FNAC) and core needle biopsy (CNB) are widely recommended for breast lesion diagnosis [14]. However, previous studies revealed that percutaneous biopsies may lead to spreading of malignant breast cells following the needle tract or hematogenous spillage [15, 16], whereas other studies reported that displaced malignant cells might not be viable due to the immune system [17, 18]. Although several case studies reported the recurrence of breast cancer in the needle tract [19], cohort studies have not found any association between local recurrence or overall survival in breast cancer patients [20, 21]. However, there seems to be a prevalent belief among patients that percutaneous biopsies might promote the spread of cancer [22]. In addition, there exists a false negative rate for FNAC and CNB due to the experience of surgeons and pathologists and the small volume of breast lumps [23, 24]. Moreover, the waiting time for paraffin pathology diagnosis after biopsies is too long, which restricts its application to some extent. Therefore, excision biopsy might be performed when other percutaneous biopsies are not feasible. When excision biopsy was performed for the intraoperative pathological assessment before SLNB, the lymphatic drainage of SLNs might be cut off, potentially leading to identification failure of SLNs. Moreover, lymph flow rerouting and fatty axilla could also reduce the accumulation of radiocolloids and blue dye in SLNs [25]. Therefore, preoperative mapping of SLNs and their lymph vessels (LVs) is helpful for avoiding inaccurate dissection and consequently improving the identification rate of SLNB.

Recently, computed tomographic lymphography (CTLG), a safe technique allowing preoperative SLN navigation, has been proposed for SLN mapping in various cancers [26–28]. These high-resolution images could be used for three-dimensional reconstruction and produce favorable results for providing precise images of the SLNs and their afferent LVs with the surrounding anatomy [25]. Moreover, CTLG could be performed during routine CT scans to preoperatively screen distant metastasis for breast cancer patients, adding little costs or time to the procedure [29]. In this study, we performed CTLG to preoperatively localize SLNs and lymph vessels in patients with early breast cancer and evaluated the usefulness and accuracy of CTLG for the localization of SLNs.

## Methods

### Patients

Between June 2017 and November 2017, 27 patients with suspected breast cancer based on the results of clinical examination and imaging methods were included in this study. The median age of these patients was 51 years (range 30–65 years). In 27 patients, the lymph nodes were clinically negative. The axillary lymph nodes of patients were negative as determined through clinical physical examination and imaging examination (mammography or ultrasound). No distant metastasis of these patients was detected. Patients with pregnancy, thyroid disease, contraindication to CT or allergy to the contrast agent were excluded. The detailed patient characteristics are presented in Additional file 1: Table S1. Informed consents were obtained from the patients before the tests. The study was approved by the Ethical Committee of Qilu Hospital of Shandong University (KYLL-2016-231).

### Computed tomographic lymphography (CTLG)

**Fig. 1:**
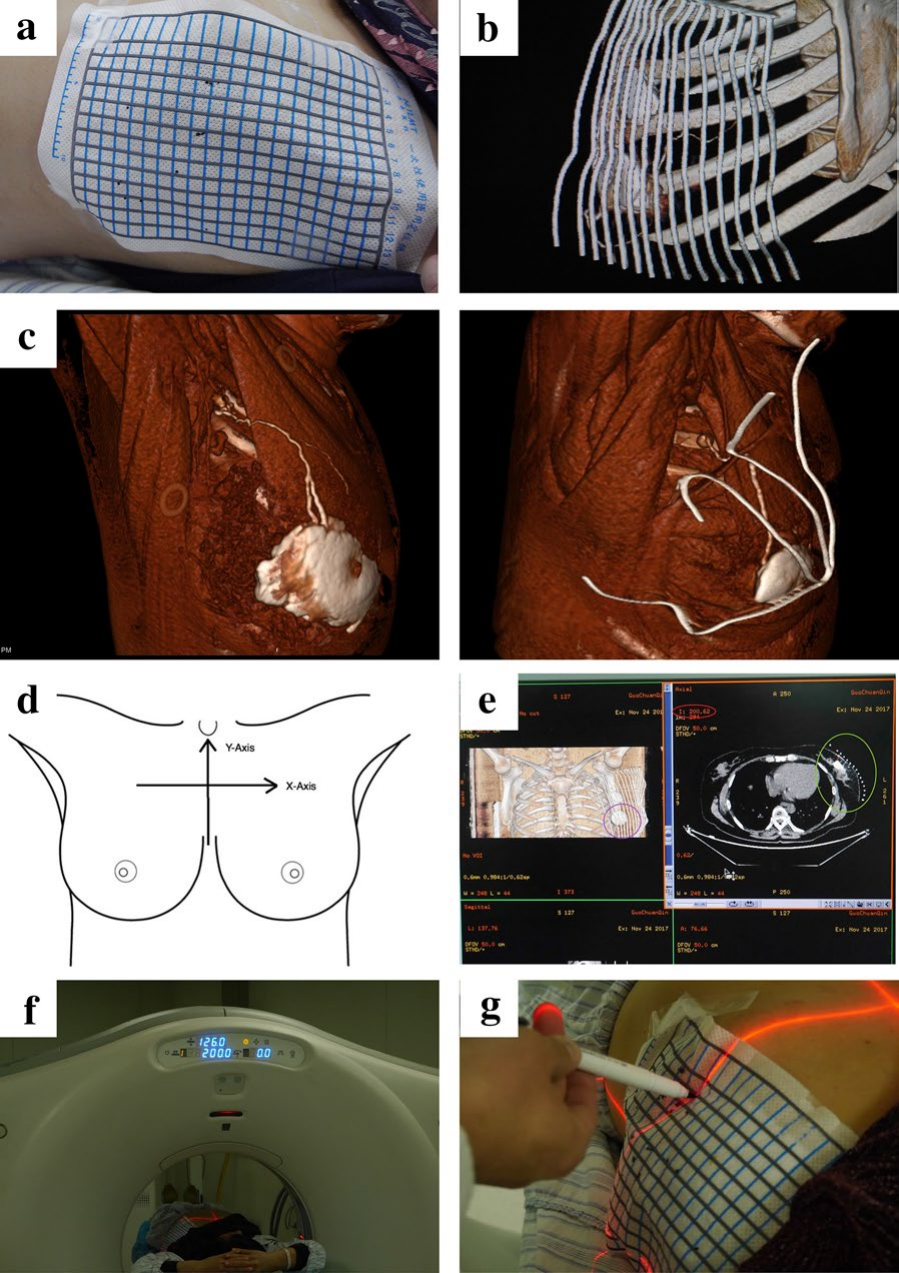
The procedure of preoperative CTLG. **a** Fix the radiopaque grid in breast and ipsilateral axilla. **b** Show the lymphatic drainage pathway using three-dimensional reconstruction of the CT scanner. **c** Representative images of three-dimensional reconstruction using SPECTRA. **d** Virtual X- and Y- axis on the human body. **e** Operation interface of CT three-dimensional reconstruction software and CT scanner. **f** Adjust the bed position to "index 200.0". **g** Localize the specific points according to X- and Y- coordinates

CTLG is a safe technique allowing SLN navigation with satisfying results, which had been proved in several cancers. Radiation exposure to patients is negligible in SLNB with CTLG, about 0.1 mSv. Patients were placed in the supine position with both arms elevated above the head and the elbows flexed. The contrast solution was composed of 10 ml iopamidol 370 (Shanghai Bracco sine pharmaceutical crop, Shanghai, China), 5 ml lidocaine hydrochloride injection, and 2.5 ml normal saline. After subcutaneous injection of 8–10 ml contrast solution in the areola and 2–3 ml contrast solution in the peritumoral area, the injection regions were messaged for 30 s. Then, the breast and ipsilateral axillary region with were covered with radiopaque grid (N. TLMT crop, Nan Jing, China) (Fig. 1a). CT was performed with a multisection scanner (SOMATOM Force 75,585, Siemens, German) by using axial scanning (120 kVp; 100 mA; slice thickness, 1.0 mm; slice interval, 0.5 mm; field of view, 512; speed, 0.75) [30]. After adjustment of color contrast and view angle, virtual 3D lymphography was obtained using the three-dimensional reconstruction software of the CT scanner or SPECTRA system, which could directly display the location of SLNs, lymphatics, and parallel lead strings attached to breast skin (Fig. 1b–c).

### Preoperative superficial localization of SLNs and lymphatics

The human body was placed in a virtual X–Y coordinate system (Fig. 1d). The lymph node or crucial point of the lymphatics is chosen in the 3D lymphography image (the red cross in purple circle, Fig. 1e) that corresponds to a point in the CT operation interface at the same location (the red cross in green circle). The index (‘I = 200.62′ in red circle), representing the sagittal position of the CT bed, is the Y-coordinate. The X-coordinate of the red cross is determined by the location relative to the parallel lead strings (the middle position of the second and third strings from medial to lateral). The X- and Y- coordinates of several crucial points, including the starting points, ending points (SLN), and inflexion points, were recorded in the same manner. Adjust the position of the CT bed based on the Y- coordinate (Fig. 1f); the red laser line cast on the human body indicates the Y-axis. The location of a specific point is at the joint point of the red laser line cast on the human body and the X- coordinate based on the radiopaque grid (Fig. 1g). The positions of all points on the body surface were marked and connected to obtain an integral lymphatic drainage pathway.

### Methylene blue and indocyanine green staining

Briefly, 0.6 ml methylene blue was injected subcutaneously, and indocyanine green was injected subcutaneously at the same point after 3 min. A near-infrared camera (Ming De, China) was used to detect fluorescent lymphatics and SLNs. SLNs were also detected following blue staining and fluorescent lymph vessels. The visualization of SLNs and lymphatics was categorized into 3 degrees, as shown in Additional file 1: Table S2. SLN++ or SLN+ was defined as successful visualization of the SLN, and LV++ or LV+ was defined as successful visualization of the LV. Successful visualization of both SLNs and LVs was defined as successful lymphography. The accuracy of CTLG was evaluated by comparing the consistency between preoperative CTLG and intraoperative detection.

## Result

### Preoperative localization of SLNs and lymphatics using CTLG

The SLNs and their LVs were successfully located in all 27 patients using preoperative CTLG with a 100% successful identification rate. Both SLNs and LVs were completely visualized (SLN++/LV++) in 19 patients. In four patients, part of the SLNs visualized, and 3 of these patients had significantly enlarged lymph nodes. There were 5 patients with only part of the LV being visualized. Of those 5 patients, the initial part was invisible in 3 patients, whereas intermediate and terminal parts were invisible in 1 patient, respectively. Both the SLN and LV were simultaneously partially visible in 1 patient. The detailed number of patients in different categories of CTLG is shown in Table 1. Certain rules exist about the courses of LVs and the connection patterns between SLNs and LVs. In the research, one LV connecting with one SLN was the most frequent pattern followed by the pattern in which one LV connects with two SLNs. Patterns in which multiple LVs connect with single or multiple SLNs were also observed; but these events were rare. The detailed connection patterns are shown in Additional file 1: Table S3. Although iopamidol was injected in both the areola and skin over the tumor, all of the observed LVs were stretched from the subareolar lymphatic plexus, flowing into SLNs, and no LV was stretched from the surface of the tumor. Detailed information about the starting points of lymphatics is presented in Additional file 1: Table S4.

**Table 1:** Number of patients in different categories of CTLG

	SLN ++	SLN+	SLN−	Overall N
LV++	19	3	0	22
LV+	4	1	0	5
LV−	0	0	0	0
N	23	4	0	27

SLN, sentinel lymph node; LV, lymph vessel

### The consistency between preoperative SLN and lymphatics localization and intraoperative detection

**Fig. 2:**
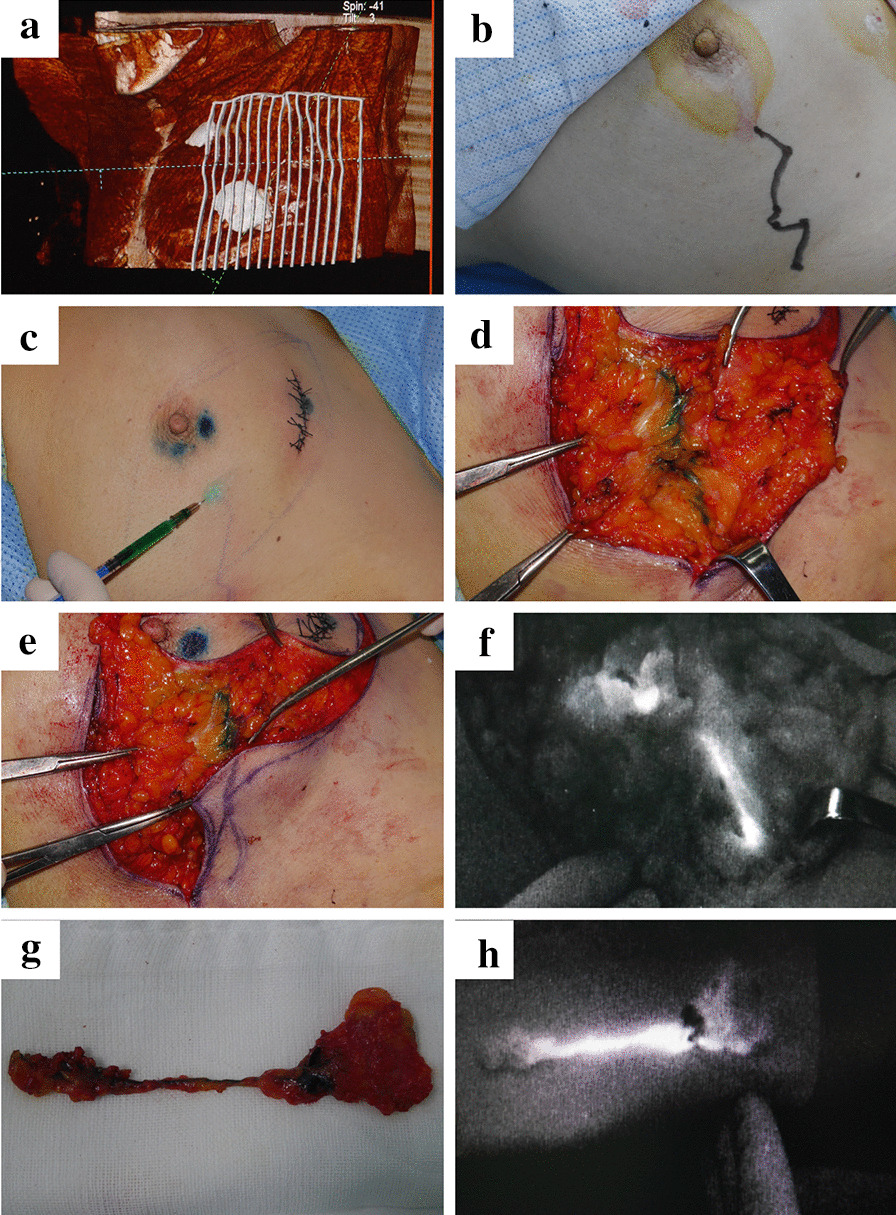
A typical case to illustrate the consistency between preoperative localization and intraoperative detection. **a** The image of three-dimensional reconstruction based on CTLG. **b** The preoperative localization of SLN and LV on body surface according to CTLG. **c** Single point injection of methylene blue and indocyanine green. **d**–**e** Comparison between preoperative localized and intraoperative blue-stained LV. **f** Comparison between preoperative localized and intraoperative fluorescent LV. **g**–**h** Comparison between blue-stained and fluorescent LV and SLN

The localization of LVs and SLNs was further verified by combined staining with methylene blue and indocyanine green fluorescence during operation. Using the result of intraoperative detection as a reference, the consistency indicates that preoperative localization revealed exactly the same course of lymphatics or completely the same trunk with few variable small branches. Moreover, the detection of more or less small branches of lymphatics, which did not connect with any SLNs, did not affect the consistency. However, different detection of the course or number of trunks by preoperative localization was considered inconsistent with intraoperative detection. A typical case that indicated the consistency between the two methods is presented in Fig. 2.

Of the 27 patients who were successfully localized through preoperative CTLG, 25 patients had consistent SLNs and LVs. The accuracy rate of SLN and lymphatic detection of preoperative CTLG was 92.6% (25/27 cases) compared with combined staining with methylene blue and indocyanine green fluorescence during operation. In total, 40 SLNs were detected using preoperative CTLG, and 42 SLNs were detected using intraoperative combined stain. The accuracy rate of SLN number detection using preoperative CTLG was 95.2% (40/42). A total of 35 lymphatics were detected through preoperative CTLG; however, more lymphatic trunks were detected during the operation in the 2 patients.

### Selection of operative incision and single point injection of stains based on preoperative CTLG

**Fig. 3:**
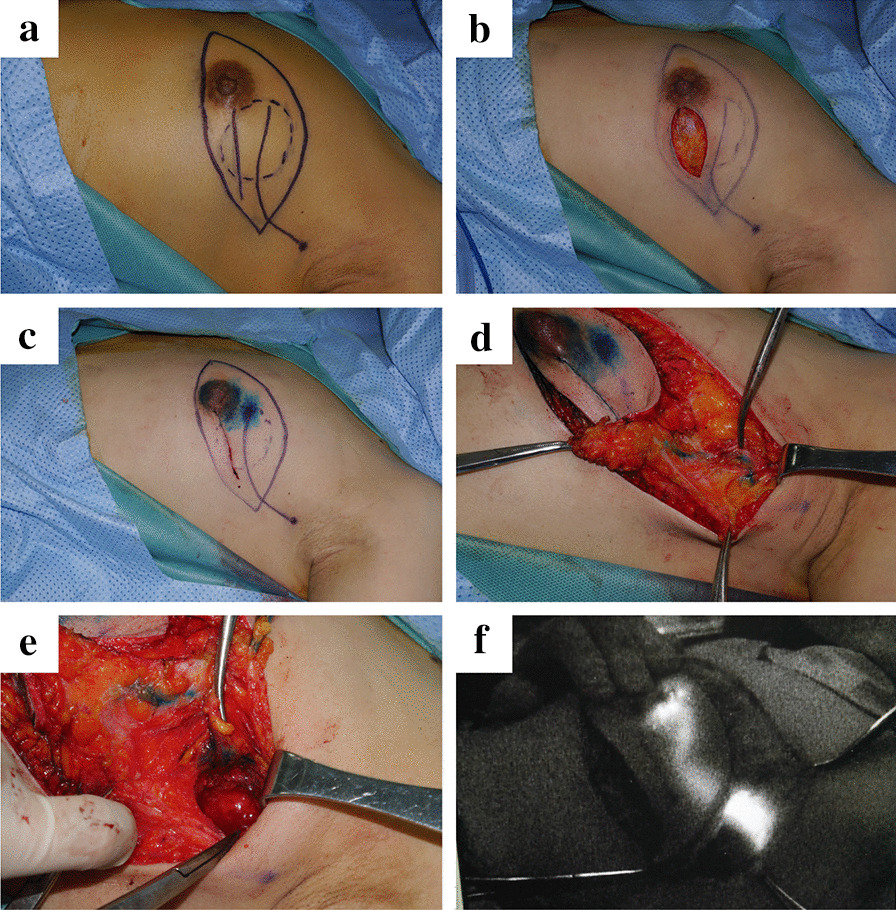
The intraoperative application of preoperative localization of SLNs and lymphatics. **a** Determine the operative incision according to preoperative localization. **b** Excise the mass far from the preoperatively localized lymphatic drainage pathways. **c** Single point injection of methylene blue and indocyanine green. **d** Explore the course of blue-stained lymphatic. **e** Localized the blue-stained SLN. **f** Detection the fluorescence after indocyanine green staining

To protect the lymphatics, an operative incision was made far from the preoperatively localized lymphatic drainage pathways (Fig. 3a–b). After tumor excision, methylene blue and indocyanine green were subcutaneously injected to a single point, namely, the initial point of lymphatics that was mapped preoperatively (Fig. 3c). In the following operation, the course of blue-stained lymphatics was carefully explored and recorded (Fig. 3d). Therefore, SLNs were found to be localized at the end of the lymphatics (Fig. 3e). Then, a near-infrared camera was used to further confirm the identity of the SLNs by detecting the fluorescence of the lymph nodes (Fig. 3f).

## Discussion

SLNB has gradually replaced ALND as the standard treatment for early-stage breast cancer patients without lymph node metastasis. SLNs are commonly identified by the combination of blue dye and radioactive tracer; however, this gold standard method is not feasible in many medical institutions, and the combination of indocyanine green and blue dye was introduced with a high identification rate for SLNs. Moreover, the reliability and safety of the SLNB procedure is dependent on the experience of surgeons [31], and even SLNB-mastered surgeons cannot avoid the identification failure of SLNs. In addition, some patients might prefer excision biopsy rather than FNAC or CNB due to the higher risk of forming hematomas or infections, cancer spreading, or the existence of false negative rate, which might cut off the LVs of the SLNs, leading to inability of dye and tracer to reach the SLNs and ultimate detection failure of SLNs intraoperatively. Therefore, preoperative localization of the lymphatic drainage pathway is helpful to improve the accuracy of SLNB.

In this study, we revealed the accurate localization of SLNs and LVs based on CTLG on the body surface of breast cancer patients, and the identification rate of SLNs was 100%, which was higher than that noted in previous studies (67–98%) [32, 33]. Moreover, the mean number of SLNs detected by CTLG was reported to be 1.1–3.1 [34], and our result was 1.5. CTLG could also help to completely understand the anatomical structure of the lymphatic pathway and the connection pattern between lymph nodes and lymph vessels, which has great potential in the study of the breast lymphatic system. Previous studies revealed that the most commonly observed LV-SLN connection pattern is one LV connecting with one SLN [33], which is consistent with our finding. In addition, CTLG information may help to predict axillary lymph node metastasis, as CTLG clearly shows enlarged lymph nodes. Although the breast was slightly enlarged due to retention of contrast agent in the connective tissue space, no other discomfort or complication occurred, and it could rehabilitate within 2–3 h after injection. Therefore, these results indicated that CTLG had satisfactory accuracy, feasibility, and safety in preoperative SLN localization.

Preoperative SLN and lymphatic localization was consistent with intraoperative detection in 25/27 patients, and all SLNs and lymphatics visualized by CTLG were detected during the operation, indicating a high accuracy of CTLG. Importantly, CTLG could also accurately locate SLNs and their lymphatics even in patients with multiple SLNs and more complex lymphatic drainage pathways. Most tumors were reported to be located in the upper outer quadrant (UOQ) of the breast [35]. As shown in our study, most of the lymphatics flowing to SLNs were also located in the UOQ of the breast. Therefore, there is a potential possibility of cutting off lymphatics when the tumor is excised, which inevitably leads to failure of SLN detection. Among the 27 patients, 3 patients had a lymphatic drainage pathway passing through or across the tumor. To avoid cutting off the lymphatic vessels, the operative incision was far from the lymphatic vessels according to the preoperative localization via CTLG. As a result, all 3 patients had complete blue-stained lymphatic vessels and fluorescent sentinel lymph nodes. Preoperative localization of SLNs could facilitate the detection of SLNs and therefore speed up the SLNB procedure [33]. Moreover, the preoperative localization of SLNs can help to reduce the anxiety of surgeons and make the identification rate less dependent on the experience of surgeons. Furthermore, there is discussion about the injection site of tracers. In some centers, periareolar injection is preferred [36], while four quadrant injections have also been reported in several studies [36]. However, satisfactory detection rates of SLNs have been reported for all injection approaches [37]. In this study, we injected the tracer at a single initial point of the lymphatic drainage pathway, which was mapped via CTLG rather than multipoint injection based on the experience of surgeons. As a result, the SLNs were all successfully detected, no complications (such as skin tattoos, pain at injection sites, local skin reactions) were observed.

Accurate distinction between SLNs and non-SLNs is significant for SLNB given that removal of non-SLNs could increase the incidence of postoperative complications, including lymphedema [38, 39], potentially due to unnecessary removal of arm reversing lymph nodes and disrupted lymphatic drainage. In this study, we found that the second-tier nodes after the SLNs were also enhanced due to the overflow of iopamidol, which may be identified as SLNs in the operation. Moreover, some LNs around the SLNs, which were not enhanced using CTLG, may also be stain blue due to the spillover effect of blue dyes, making it difficult to distinguish them from the SLNs. The exact number of SLNs that should be removed in SLNB is also controversial [40–42]. A previous study reported that a negative correlation exists between the dissected number of SLNs and false-negative rates [43], which made surgeons remove more non-SLNs to attain lower false-negative rates. One way to solve this problem is to clarify the drainage relationship between SLNs and their surrounding LNs. Our previous study reported that true SLNs were defined as LNs that first received lymphatic drainage, which could be identified with the help of precise localization of the LVs and LNs [44]. In this study, we found that CTLG could provide a good depiction of lymphatic drainage pathways. Although we found that some LNs after the SLNs were also enhanced due to the overflow of iopamidol, they were not defined as true-SLNs. Therefore, our study presents a feasible method that enables the precise identification of true-SLNs guided by lymphatic drainage pathway, contributing to accurately localizing SLNs and distinguishing true SLNs from non-SLNs, including arm reversing nodes, in breast cancer.

The distribution of iopamidol in the lymphatic system may depend on the injection volume, fluidity, and the number, size, and integrity of the associated LVs and LNs [45]. In the study, the LVs were distinctly enhanced in patients with strongly enhanced LNs, and patients with weaker LN signals also had weaker LV signals, indicating that more iopamidol would enter the LVs and LNs of individuals with higher openness of the lymphatic system. For incomplete lymphography, 3/5 cases showed absent visualization of the initial segment, 1/5 cases had an interruption in the middle segment, and 1/5 cases had no visualization of the terminal segment. The quickly passing iopamidol may lead to the absence of the initial segment, and the possible explanation for the absence of the middle and terminal segments is that a piston effect exists in lymphatic vessels [46]. Moreover, our study revealed that the time interval before lymphatic visualization was different, which may be related to the slow transportation and isolation of iopamidol in lymph node sinusoids [45]. Gentle massage of the injection site can promote the flow of iopamidol and its accumulation in SLNs [47], which is beneficial to shorten the examination time.

However, there are some limitations in our study. The number of patients is relatively small. There is no information about the adjuvant treatment and prognosis of those patients, so the prognostic value of this method cannot be quantified. Therefore, more studies are needed to evaluate the clinical value of CTLG.

## Conclusion

Our results revealed that CTLG is a feasible method for the preoperative localization of SLNs in breast cancer patients with nearly no exposure to radiation, no need for facility approval, and low cost, which could help to select the precise injection site and time of massage during SLNB and improve the identification rate of SLNs. Moreover, SLNB could be performed well for a doctor with little experience in SLNB with the help of CTLG. The use of CTLG would be a valuable complimentary tool for identifying SLNs in institutions that cannot use the radioisotope method.

## Supplementary Information

**Additional file 1: Table S1. **Characteristics of patients. **Table S2. **Category standard of CTLG. **Table S3. **Pattern of LV-SLN connection. **Table S4. **Initial point of lymphatic drainage pathway.

## Data Availability

The datasets used and analyzed during the current study are available from the corresponding author on reasonable request.

## Abbreviations

SLNB: Sentinel lymph node biopsy
CTLG: Computed tomographic lymphography
ALND: Axillary lymph node dissection
