# Supplementary material for: Application of preoperative computed tomographic lymphography for precise sentinel lymph node biopsy in breast cancer patients
Source: BMC Surg. 2021 Apr 9;21:187. doi: 10.1186/s12893-021-01190-7 (PMC8033684; doi:10.1186/s12893-021-01190-7)
Supplement: Supplementary file 1 — Additional file 1: Table S1. Characteristics of patients. Table S2. Category standard of CTLG. Table S3. Pattern of LV-SLN connection. Table S4. Initial point of lymphatic drainage pathway. [file 12893_2021_1190_MOESM1_ESM.docx]

**Additional file 1**

**Application of preoperative computed tomographic lymphography for precise sentinel lymph node biopsy in breast cancer patients**

Shishuai Wen, Yiran Liang, Xiaoli Kong, Baofeng Liu, Tingting Ma, Yeqing Zhou, Liyu Jiang, Xiaoyan Li, and Qifeng Yang

**Table S1: Characteristics of patients**

| **No.** | **Age** | **No. of SLN**  **in CTLG** | **No. of SLN**  **in operation** | **No. of positive SLN** | **No. of positive non-SLN** | **Pathological type** | **Tumor location** | **Histological grade** | **Tumor size(cm)** |
| --- | --- | --- | --- | --- | --- | --- | --- | --- | --- |
| 1 | 44 | 1 | 1 | 1 | 0 | IDC | RLI | 3 | 1.7 |
| 2 | 48 | 1 | 1 | 1 | 3 | IDC | RUO | 3 | 2 |
| 3 | 48 | 1 | 1 | 1 | 2 | IDC | RUO | 2 | 2 |
| 4 | 57 | 2 | 2 | 0 | 0 | IDC | RUO | 2 | 1.5 |
| 5 | 49 | 2 | 2 | 0 | 0 | Other | LUO | Unknown | 0.4 |
| 6 | 58 | 2 | 2 | 1 | 5 | IDC | RUO | 2 | 1.8 |
| 7 | 60 | 3 | 3 | 1 | 0 | IPC | LLO | Unknown | 3 |
| 8 | 59 | 2 | 2 | 0 | 0 | IDC | RUI | 2 | 3 |
| 9 | 51 | 1 | 1 | 1 | 7 | IDC | RUO | 3 | 2 |
| 10 | 62 | 3 | 3 | 0 | 0 | IDC | RUO | 2 | 1.3 |
| 11 | 57 | 1 | 1 | 1 | 9 | IDC | LLI | 2 | 3.2 |
| 12 | 44 | 1 | 1 | 0 | 0 | IDC | LUI | Unknown | 1 |
| 13 | 43 | 1 | 2 | 1 | 0 | IDC | LUO | 2 | 3.5 |
| 14 | 46 | 2 | 2 | 0 | 0 | IDC | LUO | 1 | 4 |
| 15 | 52 | 2 | 2 | 1 | 0 | IDC | LUI | 1 | 1.5 |
| 16 | 46 | 2 | 2 | 0 | 0 | IPC | RUO | Unknown | 1.8 |
| 17 | 64 | 1 | 1 | 0 | 0 | IDC | RUI | 3 | 3 |
| 18 | 30 | 1 | 1 | 0 | 0 | IDC | LUO | 2 | 1.2 |
| 19 | 60 | 1 | 1 | 0 | 0 | IDC | RLI | 3 | 1.7 |
| 20 | 65 | 1 | 1 | 0 | 0 | IDC | LUI | 2 | 3 |
| 21 | 49 | 1 | 1 | 1 | 0 | IDC | LLO | 1 | 3 |
| 22 | 60 | 1 | 1 | 0 | 0 | IDC | LUI | 2 | 1.7 |
| 23 | 50 | 1 | 1 | 1 | 16 | IDC | LUO | 3 | 2 |
| 24 | 55 | 1 | 2 | 0 | 0 | IDC | RUO | 3 | 1.7 |
| 25 | 42 | 2 | 2 | 1 | 5 | ILC | RUO | Unknown | 7 |
| 26 | 63 | 2 | 2 | 0 | 0 | IDC | RLO | 2 | 2.8 |
| 27 | 51 | 1 | 1 | 1 | 3 | IDC | RUO | Unknown | 2 |

CTLG, computed tomographic lymphography; IDC, Invasive ductal carcinoma; IPC, Invasive papillary carcinoma; ILC, invasive lobular carcinoma; RLI, right lower inner; RUO, right upper outer; LLO, left lower outer; RUI, right upper inner; LLI, left lower inner; LUI, left upper inner; LUO, left upper outer; RLO, right lower outer.

**Table S2: Category standard of CTLG**

| **Category** | **SLN** | **LV** |
| --- | --- | --- |
| **SLN-/LV-** | No SLN is visualized | No LV is visualized or only part of LV is visualized which is not enough to deduce the intact lymphatic pathway |
| **SLN+/LV+** | Part of SLN is visualized | Part of LV is visualized through which the intact lymphatic pathway cannot be deduced |
| **SLN++/LV++** | Complete SLN is visualized | Part of LV is visualized through which the intact lymphatic pathway can be deduced |

SLN, sentinel lymph node; LV, lymph vessel.

**Table S3: Pattern of LV-SLN connection**

| **Connection pattern** | **N** |
| --- | --- |
| 1 LV-1 SLN | 11 |
| 1 LV-2 SLN | 8 |
| 1 LV-3 SLN | 1 |
| 2 LV-1 SLN | 1 |
| 2 LV-2 SLN | 1 |
| 2 LV-3 SLN | 1 |
| 3 LV-1 SLN | 2 |
| 3 LV-2 SLN | 1 |

SLN, sentinel lymph node; LV, lymph vessel.

**Table S4: Initial point of lymphatic drainage pathway**

| **Starting point of lymphatic** | **N = 27** |
| --- | --- |
| **Right breast cancer** | 16 |
| Single starting point |  |
| 9:00-10:00 position | 2 |
| 10:00-11:00 position | 11 |
| Dual starting point |  |
| 11:00 and 1:00 position | 1 |
| Unknown | 2 |
| **Left breast cancer** | 11 |
| Single starting point |  |
| 1:00-2:00 position | 5 |
| 2:00-3:00 position | 2 |
| 6:00 position | 1 |
| Dual starting point |  |
| 1:00 and 3:00 position | 1 |
| 1:00 and 4:00 position | 1 |
| Unknown | 1 |
